# An Improved Smart Meta-Superconductor MgB_2_

**DOI:** 10.3390/nano12152590

**Published:** 2022-07-28

**Authors:** Xiaopeng Zhao, Qingyu Hai, Miao Shi, Honggang Chen, Yongbo Li, Yao Qi

**Affiliations:** Smart Materials Laboratory, Department of Applied Physics, Northwestern Polytechnical University, Xi’an 710129, China; haiqingyu@mail.nwpu.edu.cn (Q.H.); shimiao@mail.nwpu.edu.cn (M.S.); 2017100698@mail.nwpu.edu.cn (H.C.); 2014100616@mail.nwpu.edu.cn (Y.L.); qiyao@mail.nwpu.edu.cn (Y.Q.)

**Keywords:** smart meta-superconductor, p-n junction nanostructured inhomogeneous phase, electroluminescent, injecting energy, electron-surface plasmon polaritons coupling, smart superconductivity

## Abstract

Increasing and improving the critical transition temperature (*T_C_*), current density (*J_C_*) and the Meissner effect (*H_C_*) of conventional superconductors are the most important problems in superconductivity research, but progress has been slow for many years. In this study, by introducing the p-n junction nanostructured electroluminescent inhomogeneous phase with a red wavelength to realize energy injection, we found the improved property of smart meta-superconductors MgB_2_, the critical transition temperature *T_C_* increases by 0.8 K, the current density *J_C_* increases by 37%, and the diamagnetism of the Meissner effect *H_C_* also significantly improved, compared with pure MgB_2_. Compared with the previous yttrium oxide inhomogeneous phase, the p-n junction has a higher luminescence intensity, a longer stable life and simpler external field requirements. The coupling between superconducting electrons and surface plasmon polaritons may be explained by this phenomenon. The realization of smart meta-superconductor by the electroluminescent inhomogeneous phase provides a new way to improve the performance of superconductors.

## 1. Introduction

Superconductivity has greatly promoted the progress of industrial technology since its discovery, and has also expanded people’s understanding of condensed matter physics [1]. The superconducting materials have a wide range of applications, such as electric grids [2], and quantum computing devices [3,4]. The pursuit of superconducting materials with a high critical temperature *T_C_* has been promoting the research. The high-temperature superconductor [5,6], iron-based superconductor [7,8], high-pressure superconductor [9,10,11,12] and photoinduced superconductor [13,14] have been gradually studied and discovered. Superconductors have zero resistance characteristics and complete diamagnetism (the Meissner effect) [15,16,17]. Therefore, the transition from a superconducting state to a non-superconducting state has characteristic parameters: critical transition temperature (*T_C_*), critical current density (*J_C_*) and critical magnetic field (*H_C_*) [15,18]. In recent years, it has been found that the superconductivity of the sulfur hydride system is 203 K at 155 GPa [9], and the carbonized sulfur hydride system is 287.7 K at 267 GPa [12]. Although this method can achieve higher superconducting transition temperatures and even room temperature superconductivity, the extremely high pressure and small sample size limit its further applications.

The discovery of the MgB_2_ superconductor [19] has aroused great interest in the scientific community due to its excellent superconductivity and simple preparation process, especially its high *T_C_*. In order to improve the superconductivity of MgB_2_, various methods have been adopted [20,21,22,23,24], which can not only improve the practical application of MgB_2_, but also further clarify its superconductivity mechanism. Chemical doping is often used to study superconductivity. Unfortunately, many experimental results have confirmed that this approach reduces the *T_C_* of MgB_2_ [25,26,27,28]. So far, there is no effective strategy to improve the *T_C_* of MgB_2_. Chemical doping is the simplest method to change the *J_C_* of the superconductor. Doping graphene in MgB_2_ [29], and Al_2_O_3_ [30] and MgO [31] in BiSrCaCuO will reduce *J_C_* under a zero magnetic field. At the same time, adding anthracene into MgB_2_ [32] and Cr_2_O_3_ into BiSrCaCuO [33] will increase the *J_C_* under a zero magnetic field. Under a zero magnetic field, chemical doping increases or decreases *J_C_* of the superconductor, but correspondingly decreases *T_C_*. There is no particularly effective method to increase *T_C_* and *J_C_* at the same time.

Metamaterials with artificial structures have supernormal physical properties [34,35]. With the development of metamaterials, researchers proposed that a metamaterial superconductor can exhibit a higher *T_C_* [36,37,38]. In 2007, we proposed to introduce inorganic ZnO electroluminescent (EL) material into a Bi(Pb)SrCaCuO superconductor at a high temperature in order to affect its superconducting transition temperature [39,40]. In recent years, MgB_2_ and Bi(Pb)SrCaCuO smart meta-superconductors (SMSCs) have been constructed. We doped Y_2_O_3_:Eu^3+^ and Y_2_O_3_:Eu^3+^+Ag EL materials in conventional MgB_2_ and high temperature Bi(Pb)SrCaCuO superconductors to form a smart meta-superconductor [39,40,41,42,43,44,45,46,47]. When the *T_C_* of SMSCs is measured by the four-probe method, the external electric field can stimulate the inhomogeneous phase to produce EL, which can achieve the purpose of strengthening the Cooper pair and lead to the macroscopic change of *T_C_*. SMSCs is a material that can adjust and improve *T_C_* through external field stimulation, which is a new property that cannot be achieved by traditional second phase doping [44,45,46]. We believe that this is because superconducting particles acting as microelectrodes excite the inhomogeneous phase EL under the action of an applied electric field, and the energy injection promotes the formation of electron pairs. Recently, *J_C_* and the Meissner effects of the MgB_2_ and Bi(Pb)SrCaCuO smart meta-superconductors have been investigated [47]. The addition of Y_2_O_3_:Eu^3+^ and Y_2_O_3_:Eu^3+^+Ag increased the *J_C_* of MgB_2_ and Bi(Pb)SrCaCuO, and indicating the Meissner effect at higher temperatures. It has been confirmed that the rare earth oxide inhomogeneous phase can improve the *T_C_*, *J_C_*, and Meissner effect of conventional and high temperature oxide superconductors. However, it is very difficult to improve the electroluminescence intensity, short luminescence life, large applied electric field and other factors of rare earth oxides, which limits the improvement of its superconducting performance.

In this paper, the smart superconductivity of MgB_2_ was studied by introducing the p-n junction electroluminescence inhomogeneous phase to realize energy injection and improve electron pairing. Studies show that the high luminescence intensity and long life of the p-n junction nanostructure can ensure the stability of material properties. In addition, the p-n junction nanostructure excitation is easier, with only a few volts of excitation required rather than hundreds or even thousands of volts. Thus, the electric field applied by the four-point method for measuring superconductivity can be satisfied. Because the p-n junction nanostructured inhomogeneous phase exhibits good behavior under field excitation, the optimum amount of the inhomogeneous phase increased from 0.5wt.% to 1.0wt.% compared to oxide. Therefore, the critical temperature *T_C_*, current density *J_C_* and the Meissner effect of superconducting transition are higher than those of the oxide inhomogeneous phase. In particular, the performance stability of the material has been greatly improved, and can be stable for more than several hundred hours. We hold the opinion that the photons generated by the inhomogeneous phase of the p-n junction nanostructure interact with some superconducting electrons to generate surface plasmon polaritons (SPPs) and promote electron pair transport.

## 2. Model

**Figure 1:**
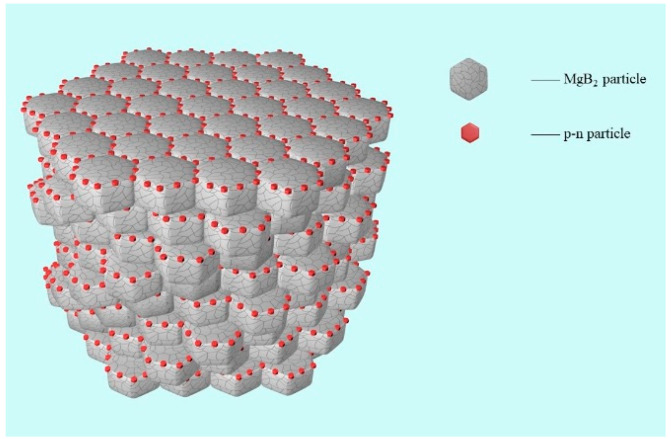
MgB_2_ SMSCs model diagram.

Figure 1 shows the MgB_2_ SMSCs model constructed with polycrystalline MgB_2_ as raw material. The gray polyhedrons are polycrystalline MgB_2_ particles, Φ is the particle size of MgB_2_ particle, which will be described in detail in the experimental part. The red particles are p-n junction particles with red light wavelengths, which are dispersed among MgB_2_ particles as the inhomogeneous phase. The introduction of the inhomogeneous phase inevitably reduces the *T_C_* of MgB_2_, mainly because the doped inhomogeneous phase is not a superconductor, which is detrimental to the superconductivity of MgB_2_, such as the MgO impurity phase in MgB_2_. For convenience, the reduction of *T_C_* after the introduction of the inhomogeneous phases is called the impurity effect [39,40,41]. The incorporation of inhomogeneous phases has been proved to be an effective method to improve *T_C_* in MgB_2_ and Bi(Pb)SrCaCuO systems. For example, the introduction of electroluminescence Y_2_O_3_:Eu^3+^ and Y_2_O_3_:Eu^3+^+Ag can produce an electroluminescence effect and increase *T_C_* [42,43,44,45,46]. There is obvious competition between the impurity effect and the EL excitation effect of the inhomogeneous phase. When the EL excitation effect is dominant, *T_C_* is improved (*ΔTc* > 0). Otherwise, the inhomogeneous phase is introduced to reduce *T_C_* (*ΔTc* < 0). Therefore, the impurity effect should be reduced as much as possible and the EL excitation effect should be enhanced in order to obtain high *T_C_* samples. The superconductivity of a smart meta-superconductor can be improved and adjusted by adding the EL inhomogeneous phase [45,46]. It has been known that variations in *T_C_* are often related to variations in electron density [48,49]. However, under the current preparation conditions, the inhomogeneous phase only exists between MgB_2_ particles and does not react with MgB_2_. Moreover, the diffusion between the inhomogeneous phase and MgB_2_ particles is difficult, and the electron density cannot be significantly changed. Therefore, electron density is not a key tuning parameter affecting *T_C_* variation. During the measurement process, the applied electric field forms a local electric field in the superconductor and excites the inhomogeneous phase to generate EL excited photon injection energy, which is beneficial to the enhancement of the Cooper pair and the change of *T_C_*. However, given that photons may destroy the Cooper pair, the mechanism for the occurrence of *T_C_* changes needs to be further explored. Later, according to the experimental results, we will explain this phenomenon by the inhomogeneous phase EL.

## 3. Experiment

### 3.1. Preparation of p-n Junction Luminescent Particles

**Figure 2:**
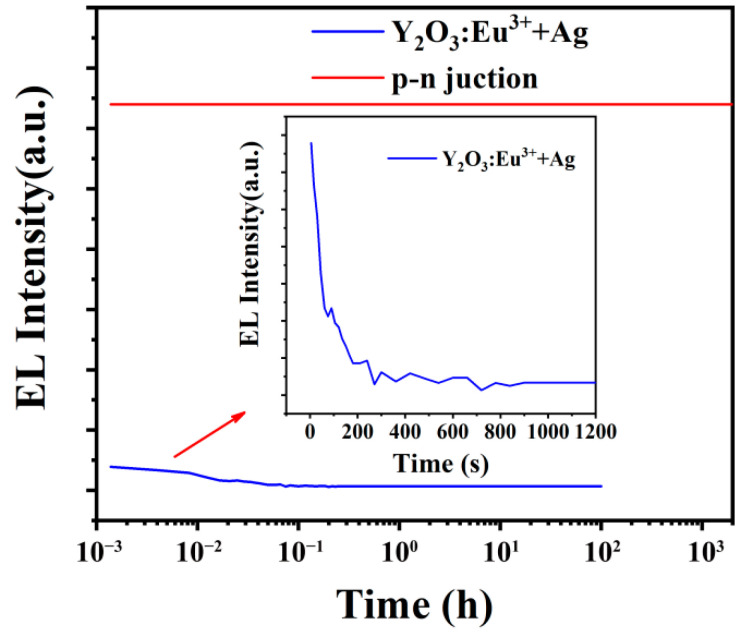
Luminescence intensity and lifetime test curves of p-n junction particles and rare earth oxide particles in red light wavelengths.

We purchased commercial red LED epitaxial chip, its luminous composition is the AlGaInP composite structure, and luminous wavelength is 623 nm. The compound AlGaInP is produced by Shandong Huaguang Optoelectronics Co., Ltd., Jinan, China. It uses trimethylgallium, trimethylindium, trimethylaluminum and phosphorane as raw materials and as reactants, which are brought into the vacuum furnace by hydrogen for reaction growth on the substrate. The melting point of the product AlGaInP is between 1200 and 1400 °C. We stripped compounds off the substrate, ground them to obtain particles of about 4 µm × 4 µm × 1.7 µm. The particles consisted of three-layer nanostructures: a p-type semiconductor layer (250 nm thick), an active layer (250 nm thick), and an n-type semiconductor layer (1200 nm thick). The electroluminescence test method is the same as that of rare earth luminescent particles, and the measurement conditions were given in the text. The applied voltage <10 V, current <10 mA, test luminescence curve is shown in Figure 2. The luminescence curves of the electroluminescent rare earth oxide particles in the figure were obtained from the samples prepared by our group [50,51]. It can be seen that the luminescence intensity of the p-n junction particles is much higher than that of electroluminescent rare earth oxide particles. After more than 2000 h of work, the luminescence intensity almost did not decay, and the luminescence behavior did not change after 100 days. The characteristics of high strength and long life of the p-n junction provided a solid foundation for improving smart meta-superconductors.

### 3.2. Preparation of MgB_2_ Superconductor and Inhomogeneous Phase Samples

**Figure 3:**
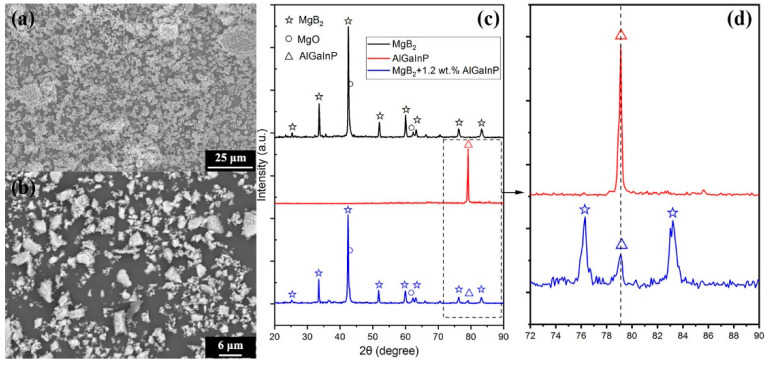
SEM diagram of (**a**) MgB_2_ particles, diameter Φ ≤ 30 μm, (**b**) p-n junction particles, ground into 4 μm × 4 μm × 1.7 μm particles, (**c**) the XRD test curves of pure MgB_2_, AlGaInP and MgB_2_ + 1.2 wt.% AlGaInP samples, (**d**) a partial enlarged drawing of (**c**).

Magnesium diboride (MgB_2_) was purchased from Alfa Aesar with a purity of 99% and a particle size of 100 mesh (150 µm). A certain amount of MgB_2_ basic powder raw material was put into a 500 mesh (30 µm) stainless steel standard sieve, and the large sized particles were removed by screening to get the basic uniform size of MgB_2_ particles, with a diameter of Φ ≤ 30 μm. Figure 3a,b show the SEM image of MgB_2_ and the p-n junction particles. Figure 3c show the XRD test curves of pure MgB_2_, AlGaInP and MgB_2_+ 1.2 wt.% AlGaInP samples. Figure 3d is a partial enlarged drawing of Figure 3c, where the vertical dotted line corresponds to the diffraction peak of AlGaInP. The comparison results show that in addition to the MgB_2_ phase, there is an independent AlGaInP phase in the doped sample, indicating that there is no chemical reaction between the two. A certain amount of MgB_2_ powder raw materials and corresponding inhomogeneous phase p-n particles with different mass fractions were weighed and put into two beakers, respectively, to make an alcohol solution and then ultrasonic was used for 20 min. The two solutions were placed on a magnetic stirrer for stirring, and the inhomogeneous phase solution was added to the MgB_2_ solution drop by drop during stirring. After the dripping, the mixed solution was stirred for 10 min and ultrasonic was used for 20 min. Then, it was transferred to petri dishes and dried in a vacuum drying oven at 60 °C for 4 h to obtain a black powder. The powder was fully ground and pressed into a wafer with a diameter of 11 mm and a thickness of 1.2 mm. The pressure and holding time were 14 MPa and 10 min, respectively. The wafer was then placed in a small box made of tantalum, and the box was then placed in an alumina porcelain boat, which was finally transferred to a vacuum tube furnace. In the high pure Ar atmosphere, the samples were slowly heated to 840 °C in the vacuum tube furnace for 10 min, then cooled to a 650 °C temperature calcination for 1h, and then slowly cooled to room temperature to obtain the corresponding samples [43,46]. Pure MgB_2_ samples (represented by S0) and the p-n junction doped MgB_2_ samples were prepared, with the concentration of dopant corresponding to each sample as shown in Table 1. In the experiment, the influence of the inhomogeneous phase of luminescence on the superconducting transition temperature of the MgB_2_-based superconductor was studied by changing the content of the inhomogeneous phase.

**Figure 4:**
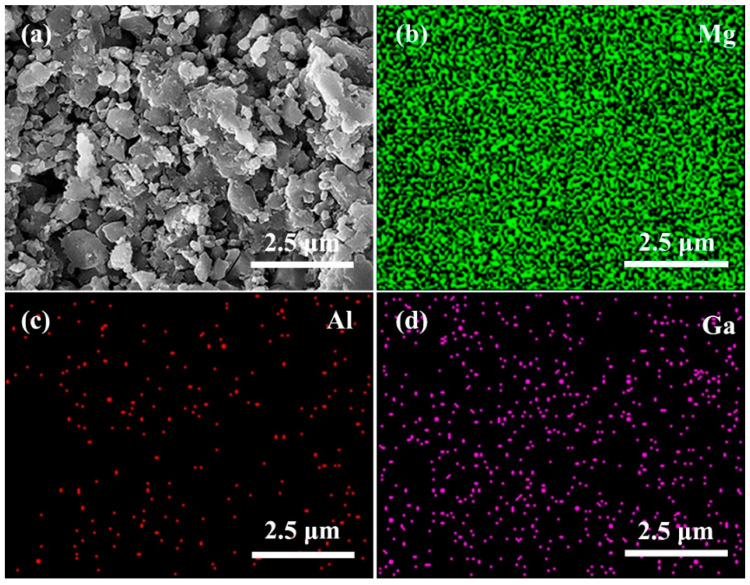
(**a**) SEM diagram of sample after sintering, (**b**–**d**) EDS mapping of Mg, Al, Ga.

Figure 4a shows the SEM image of the MgB_2_ + 0.9 wt.% p-n junction after sintering. Figure 4b–d show the EDS mapping for elements Mg, Al, and Ga listed in the top right corner of each figure. The distribution of elements in Figure 4 shows the discrete distribution of the Al and Ga elements and the aggregation distribution of the AlGaInP inhomogeneous phase. Due to the uneven size and distribution of the AlGaInP particles, as well as the distribution of Al and Ga elements in the particles during the p-n junction, preparation by vapor deposition may lead to the change of their positions and concentrations.

### 3.3. Critical Transition Temperature Measurement

A four-lead method was used to measure the *R-T* curve of the sample at a low temperature, with a distance of 1 mm between the four probes to determine the superconducting transition temperature *T_C_* of the sample. The closed-cycle cryostat manufactured by Advanced Research Systems provides a low temperature environment (the minimum temperature is 10 K); the test current (1–100 mA) is provided by the high temperature superconducting material characteristic test device, produced by the Shanghai Qianfeng Electronic Instrument Co., Ltd., Shanghai, China, Voltages were measured using a Keithley nanovolt meter; we adjusted the test temperature with a Lake Shore cryogenic temperature controller. The whole testing process was carried out in a vacuum environment [41,46].

### 3.4. Measurement of Critical Current Density and Meissner Effect

The sample was placed in a low-temperature medium and the current-voltage (*I-V*) characteristic curve was measured by a four-probe method under a zero magnetic field. A certain amount of direct current was connected to the prepared sample by two leads, and the other two leads were used to measure the voltage of the prepared sample by a Keithley digital nanovoltmeter. We used indium wire to connect the sample to the lead, and the distance between the two voltage leads for all samples is 1 mm. When the current *I* passing through the sample exceeds a certain value, the superconducting state is destroyed and changes to the normal state. This current is called the critical transport current of the superconductor. Typically in superconducting systems, the transport critical current density (*J_C_*) is determined by *I-V* measurements at different temperatures (below the initial transition temperature *T_C,on_*), with a voltage criterion of 1 μV/cm [47,52,53,54]. The shape and size of all samples and the distance between the current and voltage leads were kept the same during the test. Subsequently, the prepared samples were tested for DC magnetization [47,55]. The samples were cooled slowly in a magnetic field of 1.8 mT parallel to the plane, and data were collected during heating. All samples showed complete diamagnetism.

## 4. Results and Discussion

**Figure 5:**
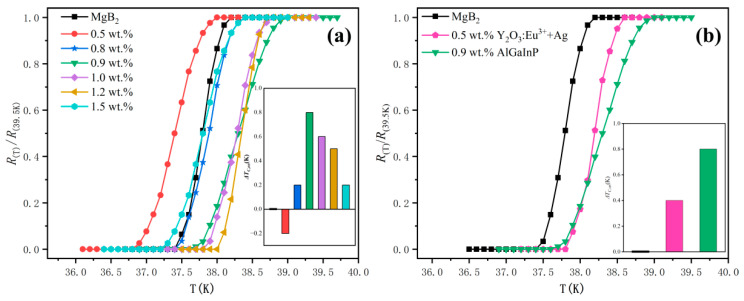
*R-T* curve of MgB_2_ sample. (**a**) Doping results of pure MgB_2_ and inhomogeneous phase at different concentrations, Insets: the values of *ΔT_C_* (*ΔT_c_* = *T_c_* − *T_cpure_*). (**b**) Comparison of the maximum variation of *∆T_C_* produced by pure MgB_2_ and oxide electroluminescence inhomogeneous phase [46], p-n junction inhomogeneous phase.

Figure 5 is the normalized resistivity curve of the doped x wt.% luminescent inhomogeneous phase p-n junction (x = 0, 0.5, 0.8, 0.9, 1.0, 1.2, 1.5) prepared with MgB_2_ raw material. x is the doping concentration, where x = 0 is the pure sample MgB_2_. The black curve in Figure 5a is the normalized *R-T* curve of the pure MgB_2_ sample, and the results show that the *T_C_* of the pure MgB_2_ sample is 37.4–38.2 K. The other six curves correspond to the *R-T* curve of MgB_2_ sample doped with the p-n junction, and the results show that the *T_C_* corresponding to these six doped samples are 36.8–38 K, 37.4–38.4 K, 37.6–39 K, 37.8–38.8 K, 38–38.7 K and 37.2–38.4 K, respectively. The test results show that at low dopant concentrations, such as 0.5 wt.%, the inhomogeneous phases reduce the *T_C_* of the MgB_2_ samples (∆*T_C_* < 0) [56,57]. However, when the dopant concentration reaches a certain value, such as 0.8 wt.%, the inhomogeneous phase enhancement effect occurs, and *T_C_* exceeds the pure sample (∆*T_C_* >0). When the dopant concentration is 0.9 wt.%, ∆*T* reaches an increased maximum value of 0.8 K and continues to increase the content of the inhomogeneous phase, while ∆*T* decreases instead. The characteristics are the same as those of the previous oxide inhomogeneous phase doping results [43,46].

**Figure 6:**
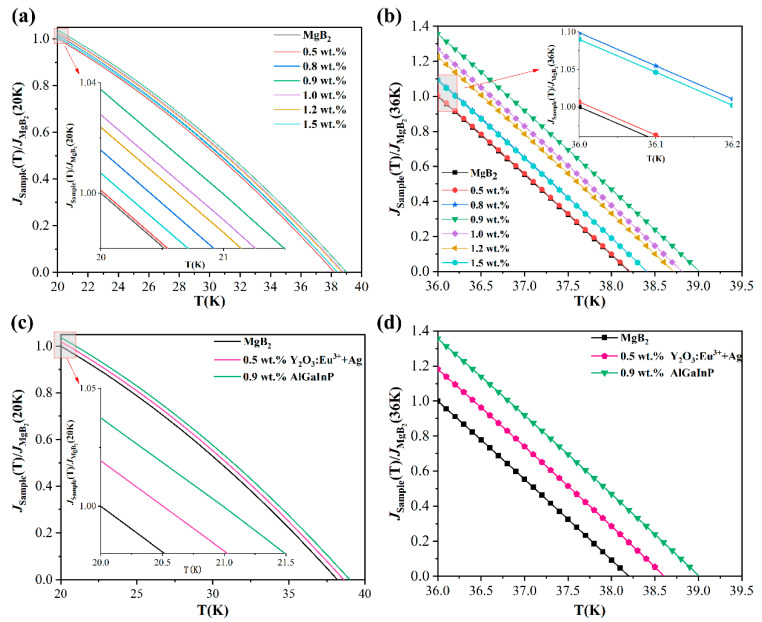
Relationship between *J_C_* and temperature *T* of MgB_2_ samples. (**a**,**b**) Pure MgB_2_ and inhomogeneous phase samples. (**c**,**d**) Comparison of *J_C_* for the maximum variation of *T_C_* produced by pure MgB_2_ and oxide luminescent inhomogeneous phase [46], p-n junction inhomogeneous phase.

Figure 6 shows the relationship between *J_C_* and the temperature of the pure MgB_2_ and p-n junction with different doping concentrations, which is determined by *I-V* measurement. It can be seen from Figure 6a that *J_C_* of pure MgB_2_ and the doped samples decreases with the increase of temperature, which is consistent with the results in literature [34,58,59]. *J_C_* of pure MgB_2_ is 8.5 × 10^4^ A/cm^2^ at 20 K, which is comparable to literature [60,61]. At this time, the *J_C_* of the 0.9 wt.% luminescent inhomogeneous phase doped sample is 8.83 × 10^4^ A/cm^2^. When the temperature is low, *J_C_* decreases slowly, and with the increase of temperature, the speed accelerates. The doping of the electroluminescence inhomogeneous phase increases *J_C_*, when *T* = 36 K, *J_C_* of samples with doping concentration of 0.9 wt.% is 37% higher than that of pure MgB_2_. When the inhomogeneous phase concentration is 0.5 wt.%, *J_C_* of the sample decreases to a minimum value faster than pure MgB_2_; and the samples with higher inhomogeneous phase concentration can have *J_C_* at a higher temperature. For example, *J_C_* of pure MgB_2_ was reduced to a minimum at 38.2 K, *J_C_* of the 0.9 wt.% doped sample was reduced to a minimum at 39 K, and *J_C_* of the 1.2 wt % doped sample was reduced to a minimum at 38.7 K.

**Figure 7:**
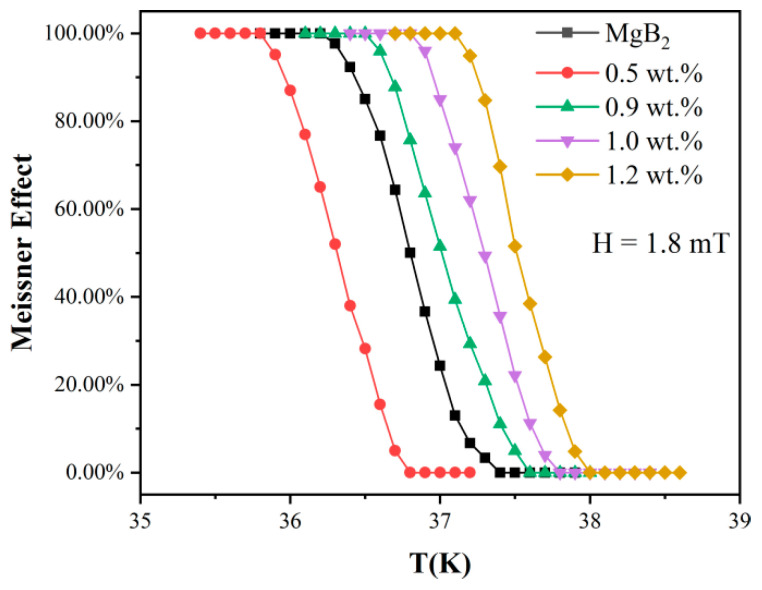
DC magnetization data of pure MgB_2_ and MgB_2_ doped with inhomogeneous phase.

Figure 7 shows the DC magnetization data of pure MgB_2_ and MgB_2_ mixed with the inhomogeneous phase. The vertical axis shows the complete diamagnetism change of the material, and the Meissner effect of all samples can be observed through the DC magnetization data. With the increase of temperature, complete diamagnetism, or the Meissner effect weakens and eventually disappears, which is consistent with literature [62,63,64]. The Meissner effect of pure MgB_2_ samples disappeared at 37.4 K, and that of the 0.5 wt.% inhomogeneous phase MgB_2_ samples disappeared at 36.8 K. The Meissner effect of 0.9 wt.%, 1.0 wt.% and 1.2 wt.% MgB_2_ samples disappeared when the temperature was higher than 37.6 K, 37.8 K and 38 K, respectively. It can be seen that the diamagnetic property of the inhomogeneous phase samples with higher concentration is greatly improved compared with that of pure MgB_2_. For example, the Meissner effect of the 0.9 wt.% and 1.2 wt.% of the inhomogeneous phase doped MgB_2_ samples was increased, compared with that of pure MgB_2_. The superconductivity of MgB_2_ doped with 1.5 wt.%, 0.9 wt.%, 1.0 wt.%, 1.2 wt.% AlGaInP were similar. Because the superconductivity of samples with the concentrations of 1.5 wt.% and 0.8 wt.% are very close, they are not marked here.

It can be seen that the p-n junction inhomogeneous phase can produce exactly the same effect as the rare earth oxide inhomogeneous phase. Figure 5b critical transition temperature and Figure 6c,d critical current intensity indicate that the p-n junction inhomogeneous phase’s behavior is further improved compared with that of the rare earth oxide inhomogeneous phase. The main reasons may be:

(1) **The advantages of the inhomogeneous phase of the p-n junction nanostructure**

**Figure 8:**
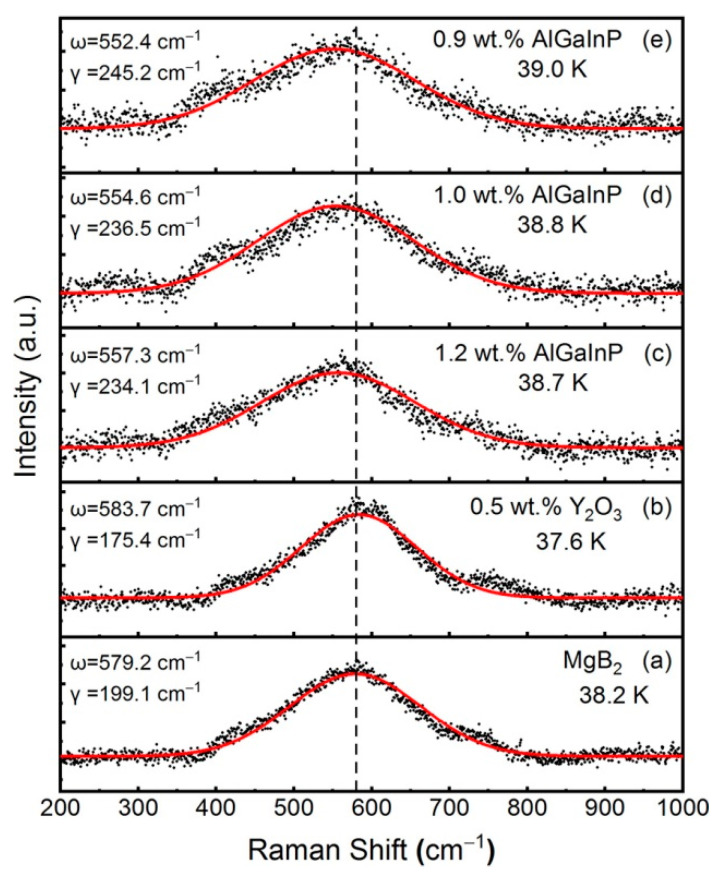
Raman spectra of (**a**) pure MgB_2_ and MgB_2_ doped with (**b**) 0.5 wt.% Y_2_O_3_, (**c**) 1.2 wt.% AlGaInP, (**d**) 1.0 wt.% AlGaInP, and (**e**) 0.9 wt.% AlGaInP.

Compared with the previous yttrium oxide inhomogeneous phase, the luminescence intensity and long life of the p-n junction nanostructure are far more than those of the previous electro-induced rare earth luminescence materials (Figure 2), which can ensure the stability of material properties. In addition, the p-n junction nanostructure excitation is easier, with only a few volts of excitation required, not hundreds or even thousands of volts. Thus, the electric field applied by the four-point method for measuring superconductivity can meet the requirement. Recently, the mechanism for increasing *T_C_* has been further explored. Figure 8 shows the Raman spectra for pure MgB_2_ and MgB_2_ doped with 0.5 wt.% Y_2_O_3_, 1.2 wt.% AlGaInP, 1.0 wt.% AlGaInP, and 0.9 wt.% AlGaInP. Y_2_O_3_ is a control dopant with no electroluminescent effect. The black scattered points are the measured data, which can be fitted by the Gaussian equation [65] and the results are shown as the red solid lines. Figure 8a show that the Raman shift (*ω*) of the main peak in the Raman spectrum of pure MgB_2_ is 579.2 cm^−1^, which corresponds to the *E*_2g_ phonon mode in MgB_2_, and the linewidth (*γ*) is 199.1 cm^−1^. The measurements are consistent with the results reported in other literature [66,67,68,69]. After doping with 0.5 wt.% Y_2_O_3_, the *ω* and *γ* of the *E*_2g_ phonon mode are 583.7 cm^−1^ and 175.4 cm^−1^ as shown in Figure 8b. The results show that the doping of Y_2_O_3_ leads to the hardening of the *E*_2g_ phonon mode [70,71,72], which weakens the electron-phonon coupling in the MgB_2_ and decreases the *T_C_* to 37.6 K. In contrast, the doping of AlGaInP leads to a decrease of *ω* and an increase of *γ*. Such a phenomenon of softening of the *E*_2g_ phonon mode indicates an enhancement of the electron-phonon coupling in the sample [66,73,74], which is beneficial to the improvement of *T_C_*. The *ω*(*γ*) for MgB_2_ doped with 1.2 wt.%, 1.0 wt.%, and 0.9 wt.% AlGaInP are 557.3 cm^−1^ (234.1 cm^−1^), 554.6 cm^−1^ (236.5 cm^−1^), and 552.4 cm^−1^ (245.2 cm^−1^), and the corresponding *T_C_* are 38.7 K, 38.8 K, and 39.0 K. *T_C_* increases with the enhancement of the softening effect. These results suggest that the softening of the *E*_2g_ phonon mode in MgB_2_ doped with AlGaInP may be the main reason for the enhancement of *T_C_*. Meanwhile, there are still some problems to be solved, such as how to obtain AlGaInP with a uniform particle size and how to distribute the AlGaInP particles evenly in the sample. We will do further research on these issues and study the mechanism in more detail in the follow-up work.

(2) **Performance improvement of smart meta-superconductor**

Because the p-n junction inhomogeneous phase exhibits good behavior under external field excitation, the optimal amount of the dopable inhomogeneous phase increases from 0.5 wt.% to 1 wt.% compared with oxide. Thus, the critical temperature *T_C_*, current density *J_C_* and the Meissner effect of superconductor can be improved compared with the oxide inhomogeneous phase, which provides a wider range for the adjustment of material properties. In particular, the performance stability of the material has been greatly improved, and can be stable for more than several hundred hours. The doping of AlGaInP with too large or too small particle sizes even decreased the *T_C_* of MgB_2_. Therefore, selecting AlGaInP with a suitable particle size is also an important factor to improve the *T_C_*. However, AlGaInP particles with a uniform size cannot be obtained by the current preparing process. Improving the preparation process to obtain AlGaInP particles with a uniform size is a focus in our follow-up work.

(3) **The origin of smart superconductivity**

**Figure 9:**
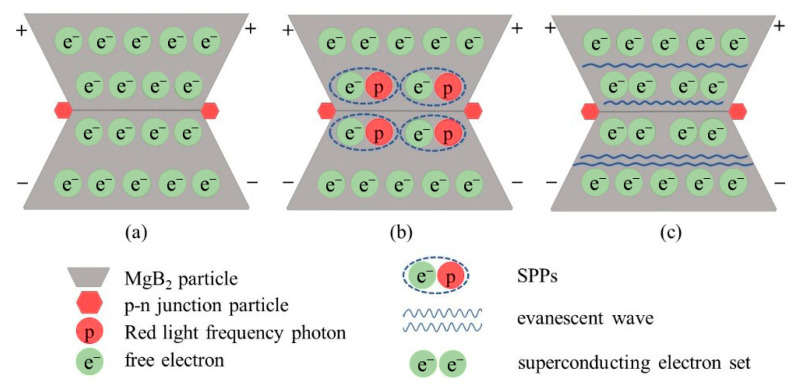
Schematic diagram of smart superconductivity generation. (**a**) Free electrons are uniformly distributed in the superconductor before the measurement field is applied. (**b**) After the measurement electric field is applied, the p-n junction particles in red light wavelength glow and generate a large number of photons. The interaction between photons and some conduction electrons occurs at the particle interface, forming plasmons. (**c**) These surface plasmons propagate as evanescent waves and can transport superconducting electrons. Due to the unimpeded transmission of evanescent wave, the superconductivity of system electrons at higher temperature is promoted.

In order to explain the above experimental results, we propose an explanatory view of smart superconductivity. Figure 9 shows the schematic diagram of smart superconductivity. By introducing the p-n junction electroluminescent inhomogeneous phase, the photon generated by the inhomogeneous phase in the external field interacts with some superconducting electrons to produce surface plasmon polaritons. The generated evanescent wave can transmit a large number of superconducting electrons with the same energy unimpeded, resulting in the surface plasma system to promote the intense interaction of electrons. The energy injection improves the electron pairing state, promote the superconductivity behavior of the material, increases the critical transition temperature, and forms smart superconductivity. 

The conventional superconductor MgB_2_ is a standard electroacoustic interaction to form a superconducting transition. It can be seen that before reaching the critical temperature *T_C_*, the current density *J_C_* (Figure 6) and the diamagnetism *H_C_* corresponding to the Meissner effect (Figure 7) of the smart meta-superconductor MgB_2_ are both higher than that of pure MgB_2_, it is due to the superposition of the electro-acoustic interaction and the SPPs interaction with superconducting electrons. In the experiment, we used two methods of heating and cooling to test. In the heating method, the temperature of the system is reduced to about 10 K first, and then the heating begins. When the temperature exceeds the critical transition temperature of the pure sample, the electro-acoustic interaction fails, but the interaction between the surface plasmas of the smart meta-superconductor and the superconducting electron still exists. Therefore, the critical transition temperature of the smart meta-superconductor MgB_2_ is higher than that of the pure MgB_2_ superconductor (Figure 5). In the state where *J_C_* of the pure MgB_2_ superconductor is zero and diamagnetism disappears, *J_C_* of the smart meta-superconductor MgB_2_ is still non-zero (Figure 6b,d) and diamagnetism still exists (Figure 7). In the cooling method, the system drops from room temperature to 39.1 K, and the initial transition occurs as the temperature continues to drop. The temperature has not reached the critical transition temperature of pure samples of 38.2 K, and the electro-acoustic effect has not yet taken place, but the superconducting transition phenomenon appears, and *J_C_* of the smart meta-superconductor MgB_2_ also appears. These phenomena confirm that electron-plasmon coupling has been formed, superconducting phase transition has occurred, and smart superconductivity has been achieved. It should be noted that, due to the presence of material resistance, when the temperature reaches a certain point, the interaction between the surface plasmas and the superconducting electrons cannot counteract the effect of material resistance, and superconductivity disappears. However, it is possible to raise the critical transition temperature further by improving the ability of the inhomogeneous phase. Since evanescent waves can exist and transmit at relatively high temperatures, the coupling of superconducting electrons with surface plasmons may promote smart superconductivity at higher temperatures.

Here, we find that the conventional superconductor MgB_2_ exhibits smart superconductivity. In fact, in previous studies, we have studied the high-temperature oxide superconductor, and the smart meta-superconductor can also be formed by doping the rare earth oxide inhomogeneous phase [44,45,47]. Therefore, it can be concluded that they can also show smart superconductivity by the p-n junction nanostructured inhomogeneous phase, and we will provide the research results in the future. 

## 5. Conclusions

In this study, by introducing the p-n junction nanostructured electroluminescent inhomogeneous phase with red wavelength to realize energy injection, the critical transition temperature *T_C_*, the current density *J_C_* and the diamagnetism of the Meissner effect *H_C_* of the smart meta-superconductor MgB_2_ are studied. The conclusions are as follows: 

(1) The smart meta-superconductor compared with pure MgB_2_, the critical transition temperature *T_C_* is increased by 0.8 K, the current density *J_C_* is increased by 37%, and the diamagnetism of the Meissner effect *H_C_* are also significantly improved.

(2) The p-n junction nanostructured inhomogeneous phase has high luminescence intensity, a long stable life and simpler external field requirements. This p-n junction nanostructured inhomogeneous phase SMSCs produces more significant performance changes than the previous yttrium oxide inhomogeneous phase. Under the same conditions, the critical transition temperature *∆T* increases by nearly 1 time, and the current density *J_C_* increases significantly. 

The coupling between superconducting electrons and SPPs is regarded as an explanation for this phenomenon. The smart meta-superconductor generated by the inhomogeneous phase opens up a new way to improve the performance of superconductors. 

## Figures and Tables

**Table 1:** MgB_2_ doped inhoumgenous phase with different concentrations.

Sample	S0	S1	S2	S3	S4	S5	S6
Inhomogeneous phase p-n junction concentration (wt.%)	0	0.5	0.8	0.9	1.0	1.2	1.5

## Data Availability

The data presented in this study are available on reasonable request from the corresponding author.
